# Variants in transient receptor potential channels and toll-like receptors modify airway responses to allergen and air pollution: a randomized controlled response human exposure study

**DOI:** 10.1186/s12931-023-02518-y

**Published:** 2023-09-07

**Authors:** Andrew Robinson, Ryan D. Huff, Min Hyung Ryu, Chris Carlsten

**Affiliations:** 1 Air Pollution Exposure Laboratory, Division of Respiratory Medicine, Department Medicine, Vancouver Coastal Health Research Institute, The University of British Columbia, Vancouver, BC Canada; 2 Channing Division of Network Medicine, Brigham and Women’s Hospital, Harvard Medical School, Boston, USA

**Keywords:** Gene-environment interaction, Allergen, TRP, TLR, Airway hyperresponsiveness, Traffic-related air pollution

## Abstract

**Background:**

Environmental co-exposure to allergen and traffic-related air pollution is common globally and contributes to the exacerbation of respiratory diseases. Individual responses to environmental insults remain variable due to gene-environment interactions.

**Objective:**

This study examined whether single nucleotide polymorphisms (SNPs) in lung cell surface receptor genes modifies lung function change and immune cell recruitment in allergen-sensitized individuals exposed to diesel exhaust (DE) and allergen.

**Methods:**

In this randomized, double-blinded, four-arm, crossover study, 13 allergen-sensitized participants underwent allergen inhalation challenge following a 2-hour exposure to DE, particle-depleted diesel exhaust (PDDE) or filtered air (FA). Lung function tests and bronchoscopic sample collection were performed up to 48 h after exposures. Transient receptor potential channel (*TRPA1* and *TRPV1*) and toll-like receptor (*TLR2* and *TLR4*) risk alleles were used to construct an unweighted genetic risk score (GRS). Exposure-by-GRS interactions were tested using mixed-effects models.

**Results:**

In participants with high GRS, allergen exposure was associated with an increase in airway hyperresponsiveness (AHR) when co-exposed to PDDE (p = 0.03) but not FA or DE. FA and PDDE also were associated with a relative increase in macrophages and decrease in lymphocytes in bronchoalveolar lavage.

**Conclusions:**

TRPs and TLRs variants are associated with increased AHR and altered immune cellularity in allergen-exposed individuals. This effect is blunted by DE exposure, suggesting greater influence of unmeasured gene variants as primary meditators of a particulate-rich co-exposure.

**Trial registration:**

The study was registered with ClinicalTrials.gov on December 20, 2013 (NCT02017431).

**Supplementary Information:**

The online version contains supplementary material available at 10.1186/s12931-023-02518-y.

## Background

Ambient air pollution exposure was associated with nearly 7 million excess deaths worldwide in 2019^1^ and increases in traffic-related air pollution and diesel exhaust (DE) exposures has contributed to increased incidence of asthma 2. Interestingly, there is a broad range in individual responses to air pollutants, a phenomenon that may be explained by underlying variation in genes that mediate cellular responses to environmental pollutants. In particular, single nucleotide polymorphism (SNP) alleles in the transient receptor potential ankyrin 1 (*TRPA1*), transient receptor potential vanilloid (*TRPV1*), toll-like receptor 2 (*TLR2*) and toll-like receptor 4 (*TLR4*) have been associated with asthma susceptibility and symptom severity 3–7.

A fraction of particulate matter (PM), specifically that under 2.5 μm in size (PM_2.5_), penetrates deeply into the lungs and is believed to be the most harmful constituent of DE 8. PM contained in DE is thought to interact with cell surface TRPs and TLRs located in lung tissues 3–7, 9. Furthermore, TRP channels expressed in the bronchioles and alveolar epithelium have been shown to increase cytokine expression, airway inflammation, nerve activation, and bronchoconstriction when activated by electrophiles and oxidants in DE 7. Likewise, the pattern recognition receptors *TLR2* and *4* expressed on human alveolar macrophages, neutrophils and the lung epithelium are implicated in inflammatory immune responses and have been shown to interact with pollution particulate matter 3, 10, 11.

As TRPs and TLRs mediate physiological responses to external stimuli in the lungs they also play a role in the pathophysiology of asthmatic exacerbation 3, 4, 6, 7. For instance, TRP channels and TLR receptors have been demonstrated to interact with allergens such as house dust mites (HDM) and Timothy grass, leading to increased cytokine expression and inflammation in the nasal and lung mucosa 12–14.

Although SNPs in TRPs and TLRs have been previously associated with asthma 3–7, further research is needed to examine if these genetic variants modify lung function change, including airway hyper-responsiveness, in response to common inhaled environmental insults. Specifically, there is a need for controlled human exposure studies to demonstrate the gene-environment interactions. The role for TRPs and TLRs in mediating immune response has been detailed for both allergen and traffic-related air pollution, such as DE 15–18. However, variants in these receptors’ coding genes and how they may enhance or blunt downstream physiology has not been carefully examined. Our group recently demonstrated that co-exposure to DE and allergen acts synergistically to promote a greater release of inflammatory mediators than allergen exposure alone, specifically increasing monocyte chemoattractant protein (MCP)-1 production and bronchioalveolar lavage (BAL) eosinophils 19. In addition, we found an impairment of lung function after an inhaled allergen challenge in 14 allergen-sensitized participants using spirometry data collected over 48 hours 20. Leveraging samples collected from this completed clinical study, we investigated whether a GRS constructed from TRP and TLR gene variants modulated lung function in response to allergen and DE co-exposure in a prospective, cross-over, direct challenge human clinical trial.

## Methods

### Participants

**Fig. 1:**
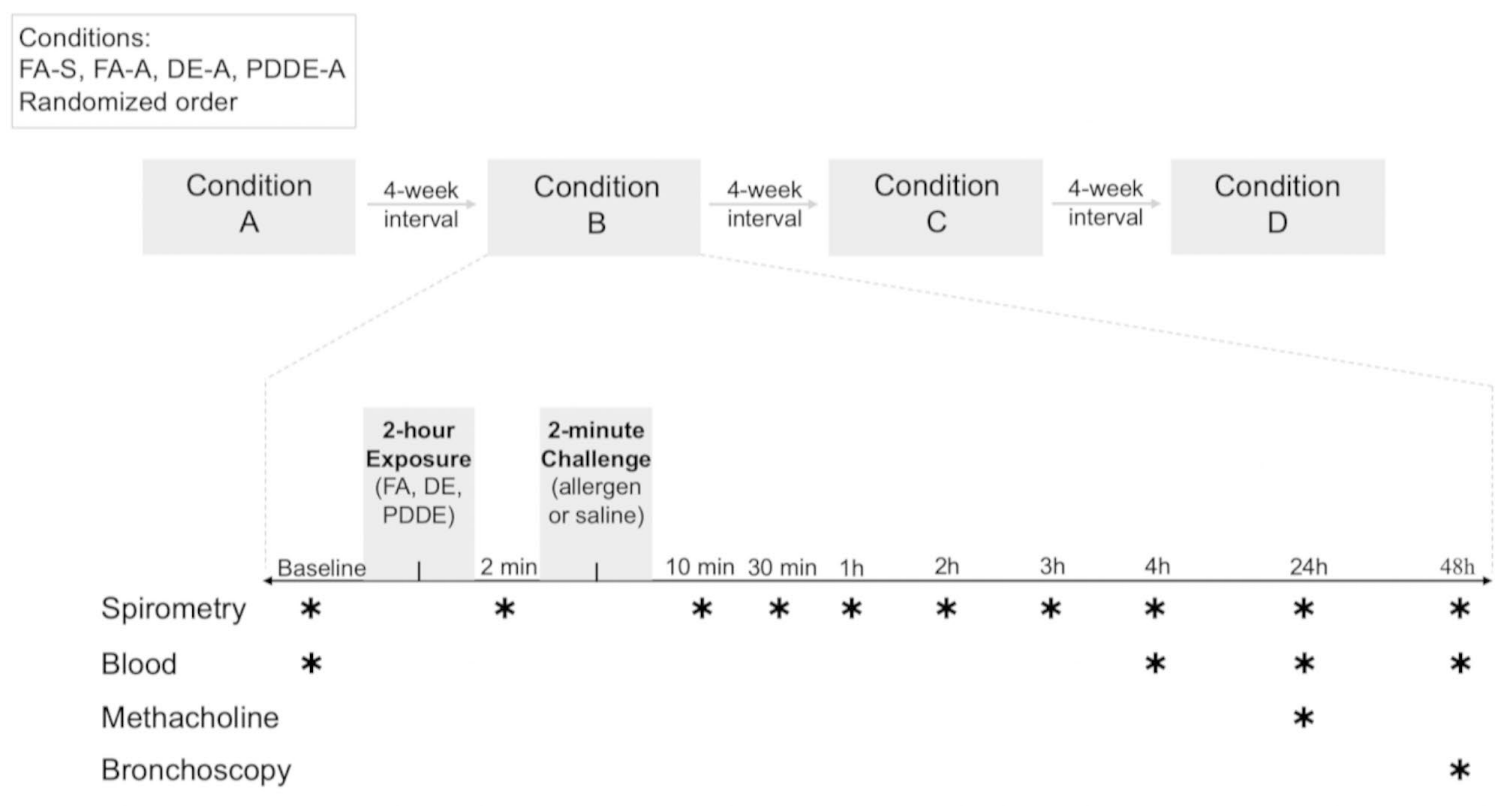
Randomized, double-blinded crossover study design. 13 participants participated in four exposure conditions. Sample collection and spirometry were performed prior to a 2-hour exposure to FA, DE, or PDDE as well as before and for 48 h after 2 min of inhaled saline or allergen challenge. Airway responsiveness (meth PC_20_) was assessed at 24-hours after exposure using a methacholine challenge. Reprinted with permission of the American Thoracic Society. Copyright © 2022 American Thoracic Society. All rights reserved. Wooding et al. 2019, Particle Depletion Does Not Remediate Acute Effects of Traffic-related Air Pollution and Allergen. A Randomized, Double-Blind Crossover Study. *Am J Respir Crit Care Med*. 200:565–574. The American Journal of Respiratory and Critical Care Medicine is an official journal of the American Thoracic Society

In a randomized, cross-over, controlled human exposure study (ClinicalTrial.gov Identifier: NCT02017431), 13 allergen-sensitized participants were recruited and exposed to four co-exposure conditions (Fig. 1 & S1) 20. The mean age of the participants was 30 ± 8 years old with 7 males and 6 females (Table 1). The co-exposures were performed in random order separated by minimum four-week washout period and were as follows: filtered air plus 0.9% saline (FA-S; negative control), FA plus allergen (FA-A), DE diluted to 300 μg/m^3^ of PM_2.5_ and allergen (DE-A), and particle-depleted DE and allergen (PDDE-A). PDDE was achieved using high-efficiency particulate absolute filtration and electrostatic precipitation which removed approximately 94% of the PM_2.5_ to an average of 18.9 μg/m^3^. In the PDDE gaseous fraction, fewer volatile organic compounds and an increase in NO_2_ was observed relative to DE 20. Participants were randomized based on computer-generated random ordering of the four different experimental conditions, and the engineers who oversaw the exposures assigned each participant to a randomized sequence. Study participants, coordinators, technicians, outcome assessor, and the study investigators were blinded to the exposure condition. Spirometry was performed before and up to 48 h after each co-exposure. Methacholine challenge was performed 24 h after allergen inhalation. Outcomes assessed for this manuscript were not the primary or secondary outcomes specified in the Clinicaltrails.gov registration 21. Please refer to the study by Wooding et al. 2019 for additional information on study recruitment, equipment, methods, and results of primary endpoints.

**Table 1:** Participant characteristics

Participant	Age (yr)	Genetic Risk Score	FEV_1_/FVC	FEV_1_% predicted	Meth PC_20_ (mg/ml)	Sex	Ethnicity	Allergen (Concentration)
1	30	11	0.89	108	> 128	M	Asian	HDM (1/32)
2	28	13	0.87	105	> 128	F	Caucasian	HDM (1/32)
3	46	13	0.78	97	> 128	F	Caucasian	Grass (1/256)
4	23	16	0.87	105	> 128	M	Caucasian	Grass (1/1,024)
5	23	9	0.69	102	0.5	M	Caucasian	Grass (1/66,538)
6	32	8	0.76	111	5.9	F	Caucasian	HDM (1/128)
7	28	10	0.78	104	2.6	M	Caucasian	HDM (1/512)
8	33	8	0.73	86	0.8	M	Caucasian	Grass (1/4,096)
9	24	15	0.74	100	0.9	F	Caucasian	HDM (1/16,384)
10	25	16	0.84	107	6.8	M	Asian	HDM (1/512)
11	23	8	0.79	85	1.6	F	Caucasian	Grass (1/256)
12	44	9	0.82	114	6.9	F	Caucasian	HDM (1/4)
13	28	9	0.87	123	3.5	M	Caucasian	Birch (1/8)
Summary	30 ± 8	11.1 ± 3.1	0.8 ± 0.06	104 ± 10	4 normally responsive, 9 hyperresponsive	7 M, 6 F	2 Asian, 11 Caucasian	7 HDM, 5 Grass, 1 Birch

*Definitions of abbreviations: HDM = house dust mite (*D. pteronyssinus*), Meth PC_20_ = Provocative concentration of methacholine causing a 20% drop in FEV_1_.

Adapted with permission of the American Thoracic Society. Copyright © 2022 American Thoracic Society. All rights reserved. Wooding et al. 2019, Particle Depletion Does Not Remediate Acute Effects of Traffic-related Air Pollution and Allergen. A Randomized, Double-Blind Crossover Study. Am J Respir Crit Care Med. 200:565–574. The American Journal of Respiratory and Critical Care Medicine is an official journal of the American Thoracic Society. Readers are encouraged to read the entire article for the correct context at [https://www.atsjournals.org/doi/full/10.1164/rccm.201809-1657OC]. The authors, editors, and The American Thoracic Society are not responsible for errors or omissions in adaptations.

### SNP genotyping

SNP genotyping was performed using DNA isolated from whole blood (QIAamp DNA Blood Mini Kit, Qiagen) using TaqMan™ SNP genotyping assays (Thermo Fisher Scientific) on a StepOne Real-Time PCR System (StepOne software v.2.3; Applied Biosystems). Positive control DNA was purchased from Coriell Institute for Medical Research (Catalogue#: HG00103, HG00581, HG00654).

The unweighted genetic risk score (GRS) was calculated from 12 single nucleotide polymorphisms (SNPs) risk alleles. The SNPs were chosen based on significant associations with air pollution and asthma development or severity in gene candidate studies or due to in vitro evidence of their increased activation by pollution particulates 3–7. Subsequently, three SNPs per gene were chosen with minor allele frequencies (MAF) greater than 0.1 based on the highest asthmatic odds ratio or highest in vitro activity to pollution particulates (Table S1). Not all SNPs were non-synonymous mutations. The risk alleles were as follows: T for rs959974 (*TRPA1*), C for rs7010969 (*TRPA1*), G for rs222747 (*TRPV1*), A for rs224534 (*TRPV1*), T for rs8065080 (*TRPV1*), C for rs3804099 (*TLR2*), A for rs4696480 (*TLR2*), G for rs2737190 (*TLR4*), C for rs10759932 (*TLR4*), T for rs1927911 (*TLR4*), and G for rs10759931 (*TLR4*). A value of 1 was assigned for each copy of a risk allele therefore giving a possible unweighted genetic risk score between 0 and 22. For rs3804099 and rs10759932, the risk allele was dominant 3, 5 and therefore a value of 2 was assigned to individuals with one or more copies of these alleles.

### Electrochemiluminescent multiplex assay

The V-PLEX Human Cytokine 30-Plex Kit (Meso Scale Diagnostics, Rockville, Maryland, USA) was used to assay interleukin (IL)-1α, IL-6, IL-8, tumour necrosis factor (TNF)-α, MCP-1, and macrophage inflammatory protein (MIP)-1β in BAL samples following the manufacturer’s protocol. See Ryu et al., 2020 for detailed methods 22.

### Statistical analysis

Statistical analysis was performed with R (version 4.1.2) in RStudio (version 2022.02.3 Build 492). Linear mixed-effects models (R package nlme version 3.1–157) were used to analyze the effect modification by GRS (exposure-by-GRS interaction) on lung function measurements, BAL immune cell proportion as well as BAL immune mediators. Participant ID were included in our model as random effects, and random intercepts were included in the models. A p-value of < 0.05 was considered statistically significant. Meth PC_20_ were log_2_ transformed and immune mediator concentrations were log_10_ transformed to satisfy the normality assumption in our models.

## Results

### Airway hyperresponsiveness increased with co-exposure to allergen and PDDE, but not DE, in those with higher GRS

**Fig. 2:**
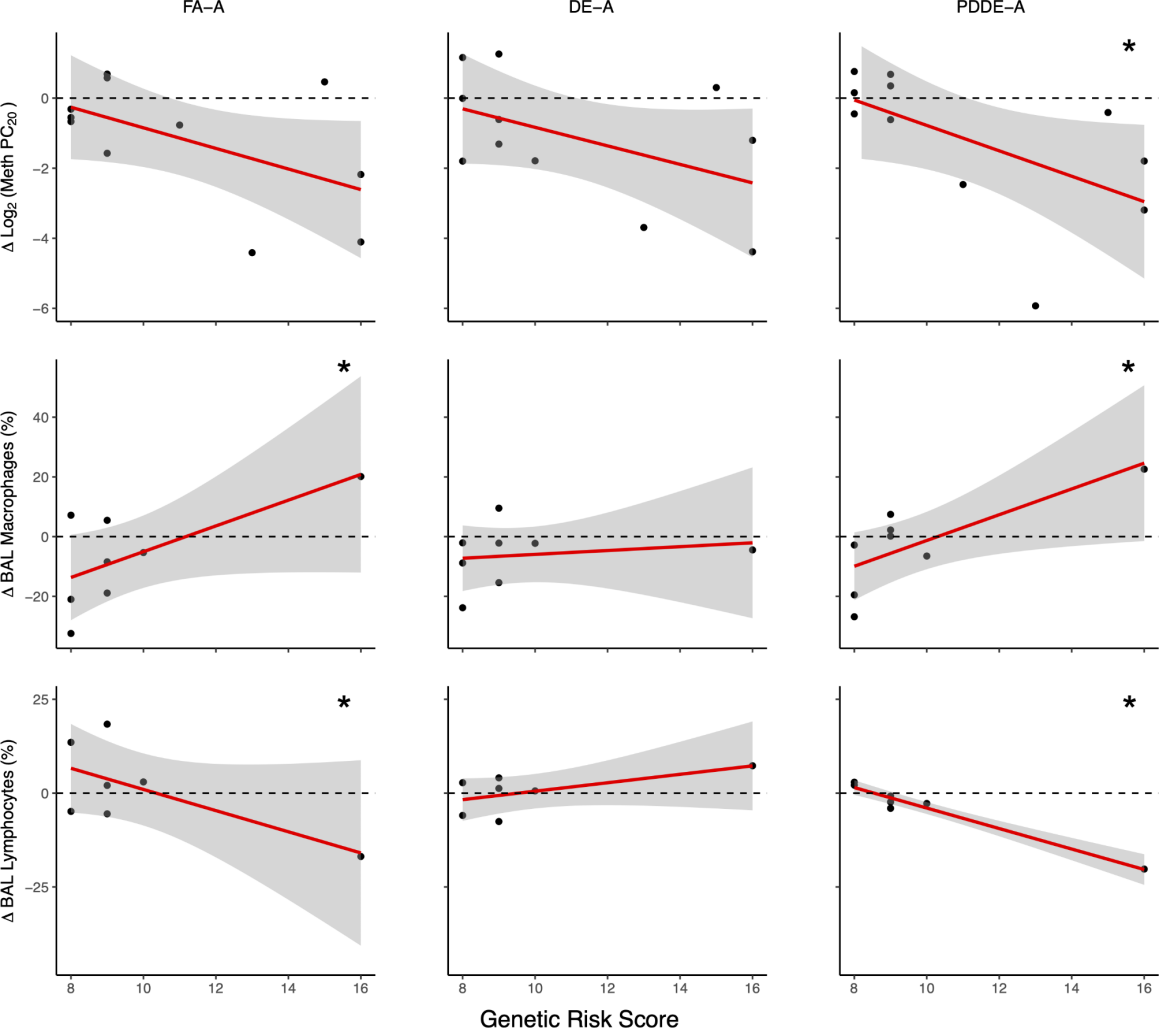
Effects of exposures and GRS on AHR (log_2_ Meth PC_20_), BAL macrophages and lymphocyte percentages. Meth PC_20_ and cell counts measured 24 and 48 h post-exposure respectively. Dashed line indicates no change from FA-S baseline mediated by the GRS or exposure conditions while points indicate individual participant’s changes from baseline. (* p < 0.05 for exposure-by-GRS interaction)

The unweighted genetic risk score was derived from 11 risk alleles in the *TLR2*, *TLR4*, *TRPA1*, and *TRPV1* genes and ranged from 8 to 16 (mean of 11.1 ± 3.1) (Table 1, Figure S2). A higher GRS theoretically indicated a dysregulation of the four receptors of interest compared to wildtype. The effect of exposure on meth PC_20_ was significantly modified by GRS for the PDDE-A condition. Specifically, participants with a higher GRS had a greater decrease in meth PC_20_ in response to allergen and PDDE co exposure (P_int_=0.03)(Fig. 2). Although not significant, the exposure-by-GRS interaction for meth PC_20_ was similar in the FA-A (*P* = 0.12) and DE-A exposure (*P* = 0.15). In order to test whether the effect modification by GRS for AHR was not driven by a single SNP, multiple linear mixed-effects models were constructed with multiple GRS each excluding one SNP. Despite the removal of each SNP from the GRS, significant exposure-by-GRS interactions were observed for meth PC_20_ except for rs224534 (*P*_*int*_ = 0.1 for PDDE-A exposure) (Table S3).

Exposure effects on FEV_1_ at 4 h in response to FA-A was significantly modified by GRS (*P* = 0.05). FEV_1_ at 24 h and forced vital capacity (FVC) at 4 and 24 h after exposure were not significantly modified by the GRS (Table S2). Furthermore, there was no significant correlation between the baseline meth PC_20_ of the FA-S condition and the GRS when compared using linear regression (*P* = 0.25, Figure S3), indicating that there was no association between GRS and AHR. Moreover, upon adjustment for both the participant’s baseline AHR and sex, the condition-by-GRS interactions remained significant (Table S4). Although, within our model AHR status modified the condition effect on log_2_ meth PC_20,_ as participants that were not AHR at baseline experienced a greater decrease in meth PC_20_. This is consistent with the findings of Wooding et al., 2019^20^.

### For those with higher GRS, co-exposure to allergen and either FA or PDDE lead to an increase in bronchoalveolar macrophages and decrease in lymphocytes

We previously reported several outcomes that were significantly altered in the BAL, in particular % eosinophils were significantly increased by allergen inhalation 19. However, in the context of GRS, the effect of exposure on BAL macrophage abundance in the airways was significantly modified for the FA-A and PDDE-A conditions (*P*_*int*_ = 0.01 and 0.01, Fig. 2.). An increase in the participant’s calculated GRS corresponded with greater increase in the percentage of lung macrophages in BAL at 48 h post-exposure. Similarly, GRS significantly modified the change in percent lymphocytes in BAL following FA-A and PDDE-A exposures (*P*_*int*_ = 0.02 and 0.02, Fig. 2.). There was no significant effect modification by GRS for macrophages or lymphocytes in the DE-A condition (*P*_*int*_ = 0.67 and 0.86, Fig. 2.) Likewise, there was no significant exposure-by-GRS interaction for bronchoalveolar recruitment of neutrophils or eosinophils in any condition relative to FA-S baseline (Table S2).

### In participants with higher GRS, exposure-induced immune mediators were reduced at 48 h in the allergen and DE co-exposure relative to the baseline FA and allergen co-exposure condition

In DE-A and PDDE-A, the effect of exposure on MCP-1 was significantly modified by GRS (*P*_*int*_ <0.01 and 0.05) as an increasing GRS corresponded with smaller accumulation of MCP-1 protein abundance in BAL. For the DE-A condition, in addition to the MCP-1, the effect of exposure was significantly modified by GRS for IL-1α, IL-6, and TNFα (*P* = 0.01, 0.04, and 0.04). An increasing GRS corresponded with less accumulation of these immune mediators at 48 h post-exposure.

## Discussion

Due to the world’s continued reliance on diesel engines and particulate-filtering technology in the transportation sector, co-exposure to DE or PDDE and an environmental allergen is commonplace. This post-hoc study provided an interesting opportunity to provide insight on potential mechanisms by which individual genotypes may be influencing exposure-response phenotypes. However, our present work is a secondary analysis not outlined in our primary design. Therefore, our report is exploratory and hypothesis-generating. Accordingly, we did not perform multiplicity adjustments. Our group has previously demonstrated that co-exposure to allergen and DE or PDDE increases AHR in individuals 20. Nevertheless, research beyond the published primary endpoints of this clinical study is warranted to elucidate individual differences in the responses to environmental insults arising from gene-environment interactions. The current study, for the first time, demonstrates that GRS constructed from SNPs in TRP and TLR genes are associated with a change in AHR and lung macrophage abundance in response to allergen-alone exposure and co-exposure of allergen and diesel exhaust with PM reduction technology applied. Furthermore, co-exposure to allergen and DE was associated with a marginal, but not statistically significant, increased AHR. Importantly, our results are consistent with a model where the effect modification by GRS on meth PC_20_ is driven primarily by exposure to allergen.

The GRS was derived from risk alleles in *TRPA1*, *TRPV1*, *TLR2* and *TLR4* that either confer an increased in vitro activity (rs222747 and rs224534) or were related to asthmatic prevalence and symptoms in gene candidate studies 3–7. In those with higher GRS, allergen-alone exposure or co-exposure to allergen and PDDE was associated with an increase in AHR and correspondingly an increase in percent macrophages and a decrease in percent lymphocytes in BAL. The TLR receptor is an important inducer of NF-κB signalling that plays a large role in generating an innate immune response 23, 24. Therefore its activation by allergen or PDDE, especially in those genetically predisposed, could explain the observed increase in macrophage recruitment. Macrophages are involved in allergic inflammation, asthma and AHR. Lung macrophages have been shown to be activated through Th2 signalling 25 or directly by allergens 26 and contribute to allergic inflammation through the production of immune mediators 26, 27. Therefore, there may be a relationship between the increase in airway responsiveness with increasing GRS and the increased recruitment of macrophages.

Activated macrophages produce IL-1α 28, MCP-1^29^ and MIP-1β 30, and IL-1α signalling causes inflammation and is upregulated during periods of oxidative stress. Likewise, MCP-1 is involved in macrophage migration and MIP-1β is needed for mounting an inflammatory immune response 29, 30. In addition to the immune mediators produced by the activated macrophages, dysregulation of the TRP channels and TLR receptors in the genetically susceptible could cause additional inflammatory cascades. Ligand-bound TLR can lead to the production of IL-1α, IL-1β, IL-6, IL-8, MCP-1 and TNF 23, 31. The activation of TRP channels can also promote TNF-α and IL-8 signalling cascades 32. A higher GRS may therefore indicate a potential importance of TRPs and TLRs in AHR upon co-exposure with allergen and either FA or PDDE.

High GRS participants showed no significant change in AHR or immune cell recruitment in the DE and allergen co-exposure. The exposure-induced production of the immune mediators IL-1α, MCP-1, IL-6, and TNFα was also reduced with increasing GRS in the DE-A condition. This is contrasted by a previous study that determined that DE and allergen co-exposure causes lung inflammation and DE exposure alone increases non-allergic inflammatory markers and MCP-1^19^. Importantly however, the activation of the *TRPV1* channel has been shown to impair MCP-1, MIP-2 and IL-6 production in macrophages leading to an anti-inflammatory effect 32, 33. Furthermore, activation of *TRPV1* on lipopolysaccharide (LPS)-stimulated macrophages has been shown to inhibit the production of additional inflammatory mediators, namely the inducible nitric oxide synthase, nitric oxide, cyclooxygenase-2 and prostaglandin 32, 34. Therefore it is plausible that the activation of *TRPV1* by electrophiles and oxidants in the DE could impair the production of MIP and MCP-1 thereby decreasing lung macrophage recruitment and consequently diminishing AHR. In other terms, the activation of *TRPV1* by DE may offset the inflammatory lung response mediated by TLR signaling, IL-1α production and macrophage recruitment. This proposed immunosuppressing effect of *TRPV1* was likely not as prominent in the PDDE and allergen co-exposure as the particle-depleting treatment removed 94% of PM_2.5_ which has both electrophilic and oxidizing properties 35 that interact with the TRP channels. An in vitro approach using air-liquid interface models may be useful in further investigating the interplay between the function of TRPV1 in modulating the inflammatory response to DE.

The current study has several limitations. Firstly, different allergens in varying concentrations were used to challenge the participants. There may be allergen-specific immune reactions that could complicate this study’s findings. Likewise, the BAL was performed at 48 h post-exposure, thereby limiting our ability to analyze more immediate changes in immune cell recruitment. The modest sample size in our study as well as interindividual variability may also be limiting. To assess potential outlier effects, we performed an analysis that removed the participant with the highest GRS score (Table S5). Results for PC_20_ PDDE-A, MCP-1 DE-A and PDDE-A, and IL-1α, IL-6, and TNFα DE-A remained significant. Furthermore, the construction of our GRS was focused on SNPs related to receptors that may directly interact with PM, however there are several genes that play an indirect role in responding to PM. These include genes in the nuclear factor κB (NF-κB), activator protein 1 (AP-1), and nuclear factor erythroid 2–related factor 2 (Nrf2) pathways that may have risk alleles that modify the effects of particulate-rich co-exposures 36, 37. Finally, there are reasons why treating each SNP equally in terms of quantifying the GRS is suboptimal, particularly due to potential linkage disequilibrium between SNPs in our GRS, however we don’t have sufficient data (such as detailed genetic ancestry) in this context to make appropriate adjustments.

## Conclusion

In summary, leveraging a controlled co-exposure to common environmental pollutants and allergens, we assessed the effect of a GRS related to lung inflammation in response to allergen and DE on lung function and cellular recruitment. We determined that increasing GRS was associated with increased AHR and macrophage recruitment when exposed to FA or PDDE and allergen. However, co-exposure to DE and allergen showed no change in AHR or immune cell recruitment and was associated with a decrease in specific immune mediators at 48 h. We theorized that *TRPV1* activation in the DE and allergen co-exposure had an immunosuppressive role. This research highlights the need to further unravel the complex interaction between underlying individual genetics and environmental exposures which lead to lung inflammation and contribute to the exacerbation of chronic lung disease.

### Electronic supplementary material

Below is the link to the electronic supplementary material.

Supplementary Material 1

Supplementary Material 2

Supplementary Material 3

Supplementary Material 4

## Data Availability

Data are available upon reasonable request. Deidentified participant data and statistical analysis code are available upon reasonable request to the corresponding author.

## Abbreviations

AHR: Airway hyperresponsiveness
AP-1: Activator protein 1
BAL: Bronchioalveolar lavage
DE: Diesel exhaust
FA: Filtered air
FEV_1_: Forced expiratory volume in 1 s
FVC: Forced vital capacity
GRS: Genetic risk score
MAF: Minor allele frequency
Meth PC_20_: Provocative concentration of methacholine causing a 20% drop in FEV_1_
NF-κB: Nuclear factor κB
Nrf2: Nuclear factor erythroid 2–related factor 2
PDDE: Particle-depleted diesel exhaust
PM_2.5_: Particulate matter with aerodynamic diameter less than 2.5 μm
SNP: Single nucleotide polymorphism
TLR: Toll-like receptor
TRAP: Traffic-related air pollution
TRPA1: Transient receptor potential ankyrin-1
TRPV1: Transient receptor potential vanilloid-1
