# Supplementary material for: Variants in transient receptor potential channels and toll-like receptors modify airway responses to allergen and air pollution: a randomized controlled response human exposure study
Source: Respir Res. 2023 Sep 7;24:218. doi: 10.1186/s12931-023-02518-y (PMC10485933; doi:10.1186/s12931-023-02518-y)
Supplement: Supplementary file 1 — Supplementary Material 1 [file 12931_2023_2518_MOESM1_ESM.docx]

***Supplementary Material***

**Variants in transient receptor potential channels and toll-like receptors modify airway responses to allergen and air pollution: A randomized controlled response human exposure study**

Andrew Robinson BSc^1*^, Ryan D Huff MSc^1*^, Min Hyung Ryu PhD^1,2^, Chris Carlsten MD, MPH^1#^

1. Air Pollution Exposure Laboratory, Division of Respiratory Medicine, Department Medicine, Vancouver Coastal Health Research Institute, The University of British Columbia, Vancouver, British Columbia, Canada
2. Channing Division of Network Medicine, Brigham and Women’s Hospital, Harvard Medical School

*First Authors.

^#^Correspondence to be addressed to:

Chris Carlsten, MD, MPH

2775 Laurel St. 7^th^ Floor, The Lung Center, Vancouver General Hospital – Gordon and Leslie

Diamond Health Care Centre, Vancouver, BC V5Z 1M9

E-mail: [carlsten@mail.ubc.ca](mailto:carlsten@mail.ubc.ca)

Telephone: 1-604-875-4729

Fax: 1-604-875-4695

Figure S1: Participant flow through the clinical trial. This study was registered with ClinicalTrials.gov (NCT02017431) and participant registration took place from Jan-2014 to Apr-2017.

Figure S2: Distribution of GRS scores. The number of patients for each GRS score was plotted.

Figure S3: Linear regression analysis of baseline airway hyperresponsiveness (meth PC_20_) in relation to GRS score.

Table S1: SNPs used in construction of the genetic risk score

| # | SNP ID | Gene | SNP Variants | Risk Allele | Risk Allele Frequency | Literature Support | Ref. |
| --- | --- | --- | --- | --- | --- | --- | --- |
| 1 | rs959974 | TRPA1 | Ref G, Alt T | T | 0.484 | OR 1.33 G/T, 1.69 T/T doctor diagnosed asthma at 7 years old | 1 |
| 2 | rs7010969 | TRPA1 | Ref A, Alt C | C | 0.632 | OR 1.43 A/C, 1.74 C/C doctor diagnosed asthma at 7 years old | 1 |
| 3 | rs222747 | TRPV1 | Ref C, Alt G | G | 0.745 | OR 2.42 C/G or G/G asthma symptom frequency, 54% greater activity to coal fly ash | 2 |
| 4 | rs224534 | TRPV1 | Ref G, Alt A | A | 0.357 | 33% greater activity for coal fly ash | 2 |
| 5 | rs8065080 | TRPV1 | Ref T, Alt C | C | 0.377 | OR 2.04 T/C poorer asthma control, OR 1.33 asthma symptom frequency | 2 |
| 6 | rs3804099 | TLR2 | Ref T, Alt C | C | 0.445 | OR 1.91 T/C or C/C asthma prevalence | 3 |
| 7 | rs4696480 | TLR2 | Ref T, Alt A | A | 0.475 | OR 1.96 T/A asthma prevalence associated with air pollution exposure | 4 |
| 8 | rs2737190 | TLR4 | Ref G, Alt A | G | 0.41 | OR 2.2 G/G asthma prevalence associated with air pollution exposure | 4 |
| 9 | rs10759932 | TLR4 | Ref T, Alt C | C | 0.116 | OR 2.9 C/T or C/C asthma prevalence associated with air pollution exposure | 4 |
| 10 | rs1927911 | TLR4 | Ref A, Alt G | A | 0.28 | OR 4.4 A/A asthma prevalence associated with air pollution exposure | 4 |
| 11 | rs10759931 | TLR4 | Ref A, Alt G | G | 0.19 | OR 2.6 G/G asthma prevalence associated with air pollution exposure | 4 |

Table S2: Effect of exposures and GRS on lung function characteristics, bronchoalveolar immune cell recruitment and BAL immune mediators.

|  | | FA-A | | DE-A | | PDDE-A | |
| --- | --- | --- | --- | --- | --- | --- | --- |
| Endpoint | Time (h) | Effect (95% CI) | P value | Effect (95% CI) | P value | Effect (95% CI) | P value |
| PC_20_ | 24 | -0.21 (-0.47 to 0.059) | 0.12 | -0.19 (-0.45 to 0.073) | 0.15 | -0.29 (-0.56 to -0.028) | **0.03** |
| FEV_1_ | 4 | 0.66 (-1.33 to 2.7) | **0.5** | -0.18 (-2.04 to 1.7) | 0.85 | 0.37 (-1.47 to 2.2) | 0.69 |
| FEV_1_ | 24 | 0.48 (-1.38 to 2.3) | 0.6 | -0.12 (-1.86 to 1.6) | 0.89 | -0.42 (-2.15 to 1.3) | 0.62 |
| FVC | 4 | -0.17 (-1.16 to 0.82) | 0.73 | -0.61 (-1.53 to 0.32) | 0.19 | -0.13 (-1.05 to 0.79) | 0.78 |
| FVC | 24 | 0.68 (-0.6 to 2) | 0.29 | 0.22 (-0.98 to 1.4) | 0.71 | 0.12 (-1.07 to 1.3) | 0.84 |
| Neutrophils | 48 | -0.21 (-0.95 to 0.54) | 0.57 | -0.43 (-1.17 to 0.32) | 0.25 | -0.17 (-0.92 to 0.57) | 0.63 |
| Lymphocytes | 48 | -3.7 (-6.62 to -0.78) | **0.02** | 0.25 (-2.67 to 3.2) | 0.86 | -3.6 (-6.49 to -0.65) | **0.02** |
| Macrophages | 48 | 4.3 (1.14 to 7.5) | **0.01** | 0.65 (-2.53 to 3.8) | 0.67 | 4.3 (1.14 to 7.5) | **0.01** |
| Eosinophils | 48 | -0.41 (-1.72 to 0.9) | 0.52 | -0.47 (-1.78 to 0.84) | 0.46 | -0.57 (-1.89 to 0.74) | 0.37 |
| IL-1a | 48 | -0.086 (-0.17 to -0.001) | **0.05** | -0.11 (-0.2 to -0.026) | **0.01** | -0.041 (-0.13 to 0.044) | 0.33 |
| IL-4 | 48 | -0.012 (-0.08 to 0.052) | 0.7 | -0.009 (-0.07 to 0.056) | 0.79 | -0.043 (-0.11 to 0.021) | 0.18 |
| IL-5 | 48 | 0.026 (-0.14 to 0.19) | 0.75 | -0.037 (-0.2 to 0.13) | 0.65 | 0.0055 (-0.16 to 0.17) | 0.95 |
| IL-6 | 48 | -0.017 (-0.07 to 0.034) | 0.51 | -0.053 (-0.1 to -0.0018) | **0.04** | -0.029 (-0.08 to 0.021) | 0.25 |
| IL-8 | 48 | 0.013 (-0.05 to 0.079) | 0.7 | -0.018 (-0.08 to 0.049) | 0.59 | 0.022 (-0.04 to 0.089) | 0.5 |
| IL-13 | 48 | 0.023 (-0.03 to 0.075) | 0.37 | -0.003 (-0.05 to 0.049) | 0.91 | 0.025 (-0.03 to 0.076) | 0.34 |
| TNF-a | 48 | 0.025 (-0.08 to 0.13) | 0.63 | -0.11 (-0.22 to -0.0044) | **0.04** | -0.056 (-0.16 to 0.051) | 0.29 |
| MCP-1 | 48 | -0.029 (-0.08 to 0.023) | 0.27 | -0.089 (-0.14 to -0.037) | **<0.01** | -0.051 (-0.1 to 0.0004) | **0.05** |
| MIP-1β | 48 | -0.027 (-0.11 to 0.052) | 0.49 | -0.074 (-0.15 to 0.005) | 0.06 | -0.038 (-0.12 to 0.041) | 0.34 |

The effect columns represent the change of the exposure relative to FA-S as modified by GRS. Cell count values are expressed as the change in percentage of cells in the BAL per GRS unit. Concentration of immune mediators were normalized using log_10_ transformation. Significant exposure effects modified by GRS (P < 0.05) are bolded.

Table S3: Effect of exposures and GRS on airway hypersensitivity (meth PC_20_). Each GRS score was constructed with the elimination of one individual SNP score.

|  | | FA-A | | DE-A | | PDDE-A | |
| --- | --- | --- | --- | --- | --- | --- | --- |
| Excluded SNP | Receptor | Effect | P value | Effect | P value | Effect | P value |
| rs959974 | TRPA1 | -0.21 | 0.10 | -0.20 | 0.10 | -0.31 | **0.03** |
| rs7010969 | TRPA1 | -0.22 | 0.10 | -0.22 | 0.10 | -0.30 | **0.04** |
| rs222747 | TRPA1 | -0.23 | 0.08 | -0.21 | 0.10 | -0.32 | **0.02** |
| rs224534 | TRPV1 | -0.14 | 0.30 | -0.13 | 0.30 | -0.21 | 0.10 |
| rs8065080 | TRPV1 | -0.19 | 0.20 | -0.16 | 0.30 | -0.29 | **0.05** |
| rs3804099 | TRPV1 | -0.29 | **0.04** | -0.26 | **0.06** | -0.37 | **0.01** |
| rs4696480 | TLR2 | -0.22 | 0.10 | -0.22 | 0.10 | -0.31 | **0.04** |
| rs2737190 | TLR2 | -0.20 | 0.10 | -0.19 | 0.20 | -0.31 | **0.03** |
| rs10759932 | TLR4 | -0.27 | 0.20 | -0.26 | 0.20 | -0.38 | **0.05** |
| rs1927911 | TLR4 | -0.22 | 0.20 | -0.18 | 0.20 | -0.31 | **0.04** |
| rs10759931 | TLR4 | -0.25 | 0.09 | -0.21 | 0.10 | -0.35 | **0.02** |

The effect columns represent the change of the exposure relative to FA-S as modified by GRS. Significant exposure effects modified by GRS (P < 0.05) are bolded.

|  | | FA-A | | DE-A | | PDDE-A | |
| --- | --- | --- | --- | --- | --- | --- | --- |
| Endpoint | Time (h) | Effect (95% CI) | P value | Effect (95% CI) | P value | Effect (95% CI) | P value |
| PC_20_ | 24 | -0.29 (-0.57 to -0.002) | **0.05** | -0.26 (-0.55 to 0.02) | 0.07 | -0.36 (-0.64 to -0.07) | **0.02** |
| FEV_1_ | 24 | -1.80 (-4.26 to 0.66) | 0.15 | -0.35 (-2.81 to 2.11) | 0.77 | -0.47 (-2.94 to 2.00) | 0.70 |

Table S4: Effect of exposures and GRS on lung function characteristics after adjustment for baseline AHR and sex.

The effect columns represent the change of the exposure relative to FA-S as modified by GRS. Significant exposure effects modified by GRS (P < 0.05) are bolded.

Table S5. Effect of exposures and GRS on lung function characteristics, bronchoalveolar immune cell recruitment and BAL immune mediators. GRS constructed with the elimination of data from the patient whose GRS was 16.

|  | | FA-A | | DE-A | | PDDE-A | |
| --- | --- | --- | --- | --- | --- | --- | --- |
| Endpoint | Time (h) | Effect (95% CI) | P value | Effect (95% CI) | P value | Effect (95% CI) | P value |
| PC_20_ | 24 | -0.21 (-0.47 to 0.059) | 0.12 | -0.19 (-0.45 to 0.073) | 0.15 | -0.29 (-0.56 to -0.028) | **0.03** |
| Neutrophils | 48 | -0.21 (-0.95 to 0.54) | 0.57 | -0.43 (-1.17 to 0.32) | 0.25 | -0.17 (-0.92 to 0.57) | 0.63 |
| Lymphocytes | 48 | -3.7 (-6.62 to -0.78) | **0.02** | 0.25 (-2.67 to 3.2) | 0.86 | -3.6 (-6.49 to -0.65) | **0.02** |
| Macrophages | 48 | 4.3 (1.14 to 7.5) | **0.01** | 0.65 (-2.53 to 3.8) | 0.67 | 4.3 (1.14 to 7.5) | **0.01** |
| Eosinophils | 48 | -0.41 (-1.72 to 0.9) | 0.52 | -0.47 (-1.78 to 0.84) | 0.46 | -0.57 (-1.89 to 0.74) | 0.37 |
| IL-1a | 48 | -0.09 (-0.17 to -0.001) | **0.05** | -0.11 (-0.2 to -0.026) | **0.01** | -0.041 (-0.13 to 0.04) | 0.33 |
| IL-4 | 48 | -0.012 (-0.08 to 0.052) | 0.70 | -0.0085 (-0.07 to 0.056) | 0.79 | -0.043 (-0.11 to 0.021) | 0.18 |
| IL-5 | 48 | 0.026 (-0.14 to 0.19) | 0.75 | -0.037 (-0.2 to 0.13) | 0.65 | 0.0055 (-0.16 to 0.17) | 0.95 |
| IL-6 | 48 | -0.017 (-0.07 to 0.034) | 0.51 | -0.053 (-0.1 to -0.0018) | **0.04** | -0.029 (-0.08 to 0.021) | 0.25 |
| IL-8 | 48 | 0.013 (-0.05 to 0.079) | 0.70 | -0.018 (-0.08 to 0.049) | 0.59 | 0.022 (-0.04 to 0.089) | 0.50 |
| IL-13 | 48 | 0.023 (-0.03 to 0.075) | 0.37 | -0.003 (-0.05 to 0.049) | 0.91 | 0.025 (-0.03 to 0.076) | 0.34 |
| TNF-a | 48 | 0.025 (-0.08 to 0.13) | 0.63 | -0.11 (-0.22 to -0.0044) | **0.04** | -0.056 (-0.16 to 0.051) | 0.29 |
| MCP-1 | 48 | -0.029 (-0.08 to 0.02) | 0.27 | -0.089 (-0.14 to -0.04) | **<0.001** | -0.051 (-0.1 to 0.00039) | **0.05** |
| MIP-1B | 48 | -0.027 (-0.11 to 0.052) | 0.49 | -0.074 (-0.15 to 0.01) | 0.06 | -0.038 (-0.12 to 0.04) | 0.34 |

The effect columns represent the change of the exposure relative to FA-S as modified by GRS. Significant exposure effects modified by GRS (P < 0.05) are bolded.

**Supplemental References**

1. Gallo V, Dijk FN, Holloway JW, et al. TRPA1 gene polymorphisms and childhood asthma. *Pediatr Allergy Immunol*. 2017;28(2):191-198. doi:10.1111/pai.12673

2. Deering-Rice CE, Stockmann C, Romero EG, et al. Characterization of Transient Receptor Potential Vanilloid-1 (TRPV1) Variant Activation by Coal Fly Ash Particles and Associations with Altered Transient Receptor Potential Ankyrin-1 (TRPA1) Expression and Asthma. *Journal of Biological Chemistry*. 2016;291(48):24866-24879. doi:10.1074/jbc.M116.746156

3. Smit LAM, Siroux V, Bouzigon E, et al. *CD14* and Toll-like Receptor Gene Polymorphisms, Country Living, and Asthma in Adults. *Am J Respir Crit Care Med*. 2009;179(5):363-368. doi:10.1164/rccm.200810-1533OC

4. Kerkhof M, Postma DS, Brunekreef B, et al. Toll-like receptor 2 and 4 genes influence susceptibility to adverse effects of traffic-related air pollution on childhood asthma. *Thorax*. 2010;65(8):690-697. doi:10.1136/thx.2009.11963
