# Supplementary figures and images for: Variants in transient receptor potential channels and toll-like receptors modify airway responses to allergen and air pollution: a randomized controlled response human exposure study
Source: Respir Res. 2023 Sep 7;24:218. doi: 10.1186/s12931-023-02518-y (PMC10485933; doi:10.1186/s12931-023-02518-y)

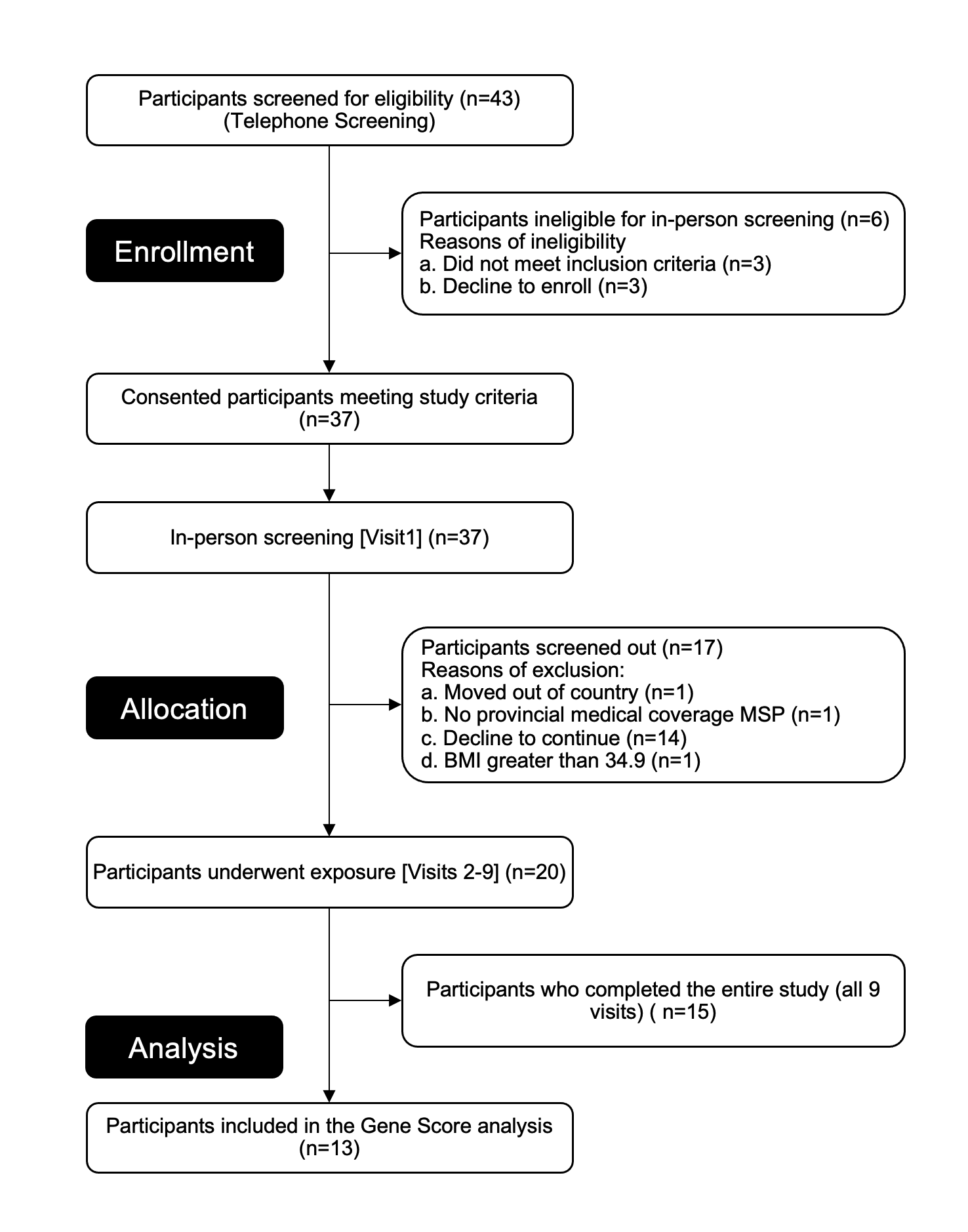

Supplement: Supplementary file 2 — Supplementary Material 2 [file 12931_2023_2518_MOESM2_ESM.png]

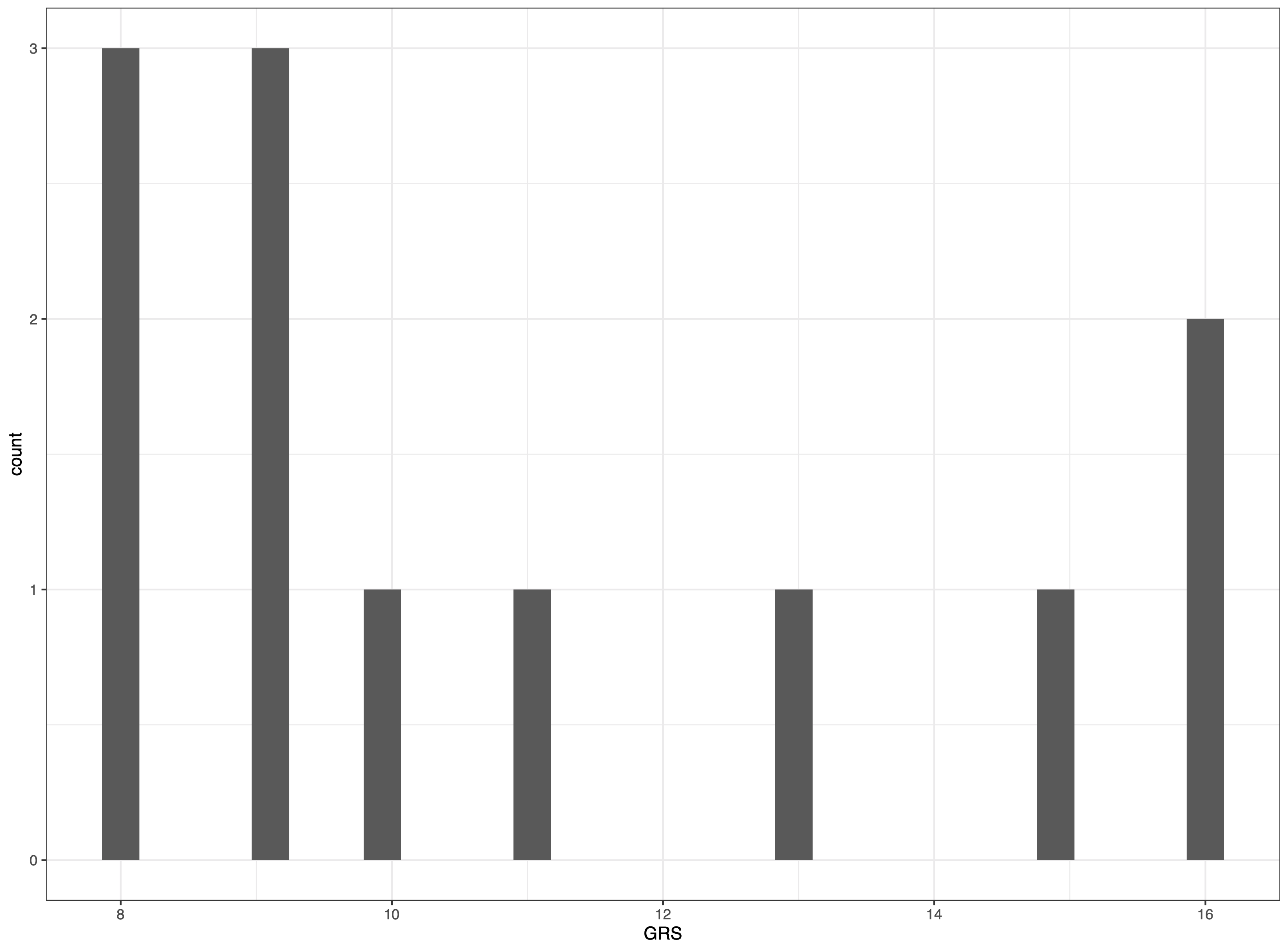

Supplement: Supplementary file 3 — Supplementary Material 3 [file 12931_2023_2518_MOESM3_ESM.png]

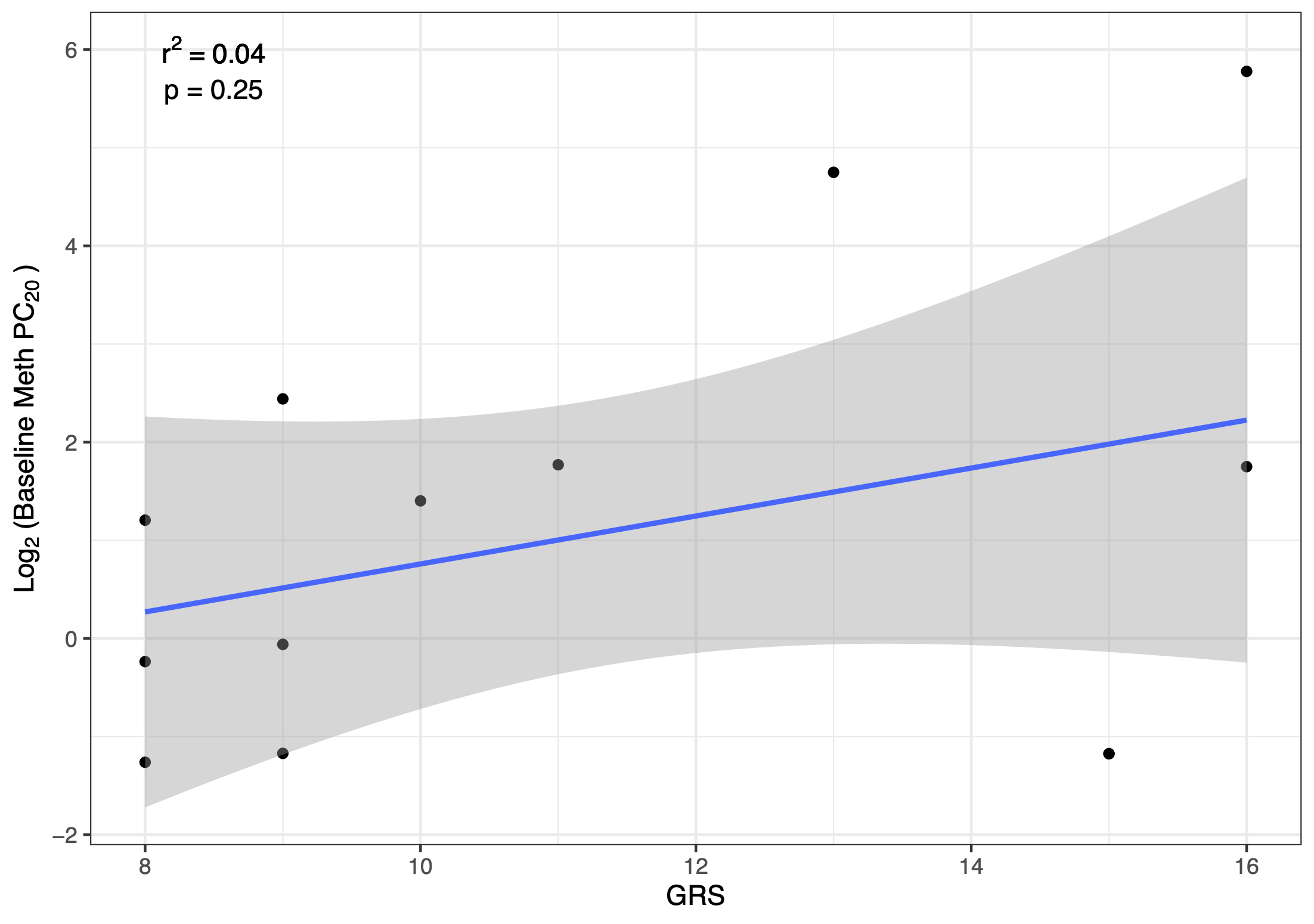

Supplement: Supplementary file 4 — Supplementary Material 4 [file 12931_2023_2518_MOESM4_ESM.png]
